# Immunity of nanoscale magnetic tunnel junctions with perpendicular magnetic anisotropy to ionizing radiation

**DOI:** 10.1038/s41598-020-67257-2

**Published:** 2020-06-23

**Authors:** Eric Arturo Montoya, Jen-Ru Chen, Randy Ngelale, Han Kyu Lee, Hsin-Wei Tseng, Lei Wan, En Yang, Patrick Braganca, Ozdal Boyraz, Nader Bagherzadeh, Mikael Nilsson, Ilya N. Krivorotov

**Affiliations:** 1 Department of Physics and Astronomy, University of California, Irvine, California 92697 United States; 2 Department of Chemical Engineering and Materials Science, University of California, Irvine, California 92697 United States; 3 Department of Chemistry, University of California, Irvine, California 92697 United States; 4 Western Digital, San Jose, California 95135 United States; 5 Department of Electrical Engineering and Computer Science, University of California, Irvine, California 92697 United States

**Keywords:** Magnetic properties and materials, Spintronics, Electronic and spintronic devices, Experimental nuclear physics

## Abstract

Spin transfer torque magnetic random access memory (STT-MRAM) is a promising candidate for next generation memory as it is non-volatile, fast, and has unlimited endurance. Another important aspect of STT-MRAM is that its core component, the nanoscale magnetic tunneling junction (MTJ), is thought to be radiation hard, making it attractive for space and nuclear technology applications. However, studies on the effects of ionizing radiation on the STT-MRAM writing process are lacking for MTJs with perpendicular magnetic anisotropy (pMTJs) required for scalable applications. Particularly, the question of the impact of extreme total ionizing dose on perpendicular magnetic anisotropy, which plays a crucial role on thermal stability and critical writing current, remains open. Here we report measurements of the impact of high doses of gamma and neutron radiation on nanoscale pMTJs used in STT-MRAM. We characterize the tunneling magnetoresistance, the magnetic field switching, and the current-induced switching before and after irradiation. Our results demonstrate that all these key properties of nanoscale MTJs relevant to STT-MRAM applications are robust against ionizing radiation. Additionally, we perform experiments on thermally driven stochastic switching in the gamma ray environment. These results indicate that nanoscale MTJs are promising building blocks for radiation-hard non-von Neumann computing.

## Introduction

**Figure 1:**
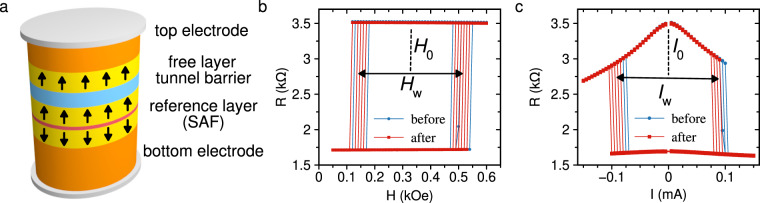
Sample schematic and switching experiments. (**a**) Schematic of a nanoscale perpendicular MTJ. (**b**) Field and (**c**) current induced spin transfer torque switching characteristics of a nanoscale MTJ before and after TRIGA irradiation. Shown in (**b**,**c**) are 10 descending and 10 ascending traces.

Spin transfer torque random access memory (STT-MRAM) is a next-generation non-volatile memory technology^1–4^ that has the advantage of fast write times^5–7^, relatively low power consumption^8–11^, and shows promise of scalability down to at least 7 nm CMOS technology node^12,13^. STT-MRAM has already found its applications in the form of stand-alone nonvolatile memory^14^, and efforts to realize embedded versions of STT-MRAM are under way^15,16^. The core component of STT-MRAM is a nanoscale magnetic tunnel junction (MTJ)^17–19^ that consists of ferromagnetic metallic layers separated by a non-magnetic insulating tunnel barrier as illustrated in Fig. 1(a). Since the MTJ does not contain semiconductor components, STT-MRAM can be radiation hard, i.e. robust to the effects of ionizing radiation. This makes STT-MRAM potentially attractive for applications in space and military technologies, particle accelerators, and nuclear reactors^20,21^. In fact previous studies have shown the promise of the radiation hardness of MTJs^22–24^; however, the effects of ionizing radiation on spin transfer torque switching in MTJs with properties required for scalable STT-MRAM technology have not been experimentally studied.

An STT-MRAM bit is written by a current pulse that applies spin torque^25^ to magnetization of the free layer ferromagnet and reverses its direction thereby changing the relative alignment of magnetic moments of the free and pinned layers of the MTJ between parallel and antiparallel^26–28^. This free layer switching leads to a change of the MTJ resistance due to the tunneling magneto-resistance (TMR) effect^28^, which allows resistive readout of the bit. The pioneering work by Ren *et al*.^22^ studied the effects of ionizing gamma and neutron radiation on micrometer-scale scale MTJs with in-plane orientation of magnetic moments of the ferromagnetic layers. This study concluded that ionizing radiation has negligible impact on TMR and field-induced switching of these MTJs. However, the MTJ devices studied were too large to be switched by spin transfer torque and thus could not be directly used in STT-MRAM. More recent work by Cui *et al*.^24^, has shown that the electric transport and magnetic properties of in-plane MTJs used in commercial toggle-MRAM^29^, which relies on switching by magnetic field, are immune to gamma radiation. However, the CMOS bit-read circuitry was found to be influenced by the radiation.

The seminal work by Hughes *et al*.^23^ demonstrated that gamma and proton irradiation had negligible effect on spin transfer torque switching of in-plane magnetized elliptical MTJs. Modern STT-MRAM is based upon nanoscale MTJs with strong perpendicular magnetic anisotropy (PMA) that forces the easy magnetization axis to be perpendicular to the plane of the sample, or so called perpendicular MTJs (pMTJs); this is due to the fact that unlike in-plane MTJs, pMTJs provide a route towards scalable STT-MRAM technology^12^. The impact of ionizing radiation on PMA has not previously been reported. PMA plays a crucial role in determining the thermal stability and critical writing currents in pMTJs, both of which are key parameters of STT-MRAM operation. As the dominant source of PMA is interfacial anisotropy, interface defects potentially induced by ionizing radiation could be a greater concern in pMTJs than in in-plane MTJs. Additionally, the effects of neutron irradiation on the spin torque switching of MTJs has yet to be performed, which is of concern due to the large concentration of boron with high inelastic neutron scattering cross section typically employed in the magnetic layers. Therefore to determine the viability of practical STT-MRAM technology for radiation hard applications, studies of the effect of ionizing radiation an spin transfer torque switching in pMTJs are required.

In this Article, we report experimental studies of the effect of extreme doses of ionizing gamma and neutron radiation on nanoscale pMTJs, whose dimensions and magnetic anisotropy are very similar to those currently employed in STT-MRAM technology. To put the ionization radiation used in this study into perspective, the largest total ionizing dose (TID) we subject the pMTJs to is 100× greater than what is required for the inter-planetary Europa Clipper Mission^30^. This mission aims to send a radiation tolerant spacecraft into orbit of Jupiter that needs to survive 40–50 flybys in the particularly severe radiation environment of the Jovian moon Europa^31^. Additionally, electronic components highly immune to neutron and gamma radiation can be used in measurement and automation systems for nuclear fuel cycle monitoring and nuclear accident decontamination^32^. Both the gamma and neutron TIDs in this study are 1–2 orders of magnitude greater than previous studies on MTJs^22–24^.

We measure the impact of radiation on several MTJ characteristics, including TMR, MTJ switching induced by magnetic field and, most importantly, MTJ switching induced by spin transfer torque. We also make *in situ* time resolved measurements of thermally activated switching of MTJs with superparamagnetic free layers in the gamma ray environment. This is of interest because MTJs with superparamagnetic free layers can serve as building blocks for non-von Neumann computation such as neuromorphic computing^33^ and invertible logic^34^. We find that high doses of ionizing radiation have negligibly small effect on all key performance metrics of nanoscale MTJs.

## Results

### Perpendicular magnetic tunnel junctions

A typical structure of a pMTJ for STT-MRAM is schematically shown in Fig. 1(a). The device consists of: (i) a free ferromagnetic metallic layer, (ii) a MgO tunnel barrier, (iii) a composite metallic ferromagnetic reference layer and (iv) top and bottom non-magnetic metallic electrodes. The ferromagnetic reference layer is typically a synthetic antiferromagnet (SAF), which consists of two ferromagnetic layers antiferromagnetically coupled to each other via an ultra-thin non-magnetic metal spacer layer^35^. Since SAF has a nearly zero net magnetic moment, it (i) minimizes an unwanted stray magnetic field from the reference layer acting upon the free layer and (ii) is stable against perturbations by external magnetic field. For practical STT-MRAM, all magnetic layers and the MgO barrier in the MTJ elements of interest are just a few Å to a few nm thick and the MTJ lateral dimensions are tens of nanometers. In this study, the magnetic free layer is a 1.6 nm thick Co_60_Fe_20_B_20_ layer. The SAF reference layer consists of

$${[{\rm{Co}}(0.4{\rm{nm}})/{\rm{Pt}}(0.5{\rm{nm}})]}_{6}/{\rm{Ru}}({d}_{{\rm{Ru}}})/{[{\rm{Co}}(0.4{\rm{nm}})/{\rm{Pt}}(0.5{\rm{nm}})]}_{4}/{{\rm{Co}}}_{60}{{\rm{Fe}}}_{20}{{\rm{B}}}_{20}(0.9\,{\rm{nm}}),$$

where square brackets indicate superlattice repetition with repetition number given by subscript, and *d*_Ru_ is the Ru layer thickness chosen to maximize antiferromagnetic coupling between the SAF magnetic layers^36–38^. We use MgO layer with resistance-area product of 10 Ω·μm^2^ corresponding to the MgO layer thickness of approximately 1.0 nm^39^. To study the effects of ionizing radiation on nanoscale MTJs, we employ both circular 60 nm (diameter) and elliptical 50 × 150 nm^2^ (minor × major axes) pMTJs fabricated on thermally oxidized silicon substrates.

### Ionizing radiation

The metallic layers of the MTJ are expected to be robust against ionizing radiation because the density of electronic states near the Fermi level in metals is high, and thus crystallographic defects induced by irradiation have little impact on conductivity. In contrast, radiation damage of the MgO barrier can potentially alter TMR and the critical current for current-induced switching of the MTJ free layer. Indeed, atomic displacement in the ultra-thin MgO layer or at the MgO/ferromagnet interfaces can induce significant modifications of the tunneling current near the defect site due to exponential sensitivity of the current to the barrier thickness and height. This, in turn, can affect both the magnitude of TMR and the current-induced switching process. Additionally, defects created at the MgO/ferromagnet interface could alter the perpendicular magnetic anisotropy in pMTJs which could affect the stability of the free layer and switching dynamics.

Gamma radiation can generate electron-hole pairs in the MgO dielectric, which can lead to dielectric breakdown of the MgO barrier if sufficiently high density of the trapped charges is reached^40^. Irradiation of an MTJ by thermal neutrons can, in principle, also induce structural damage in the MgO barrier. For example, ^10^B isotope present in the ferromagnetic layers of the MTJ has high scattering cross section for a nuclear reaction in which a ^7^Li ion and an *α*-particle are produced^41^. Depending on the path of the reaction, the *α*-particle carries kinetic energy of either 2.31 MeV or 2.79 MeV, which is high enough to create significant structural damage of the MgO barrier if the *α*-particle passes through the barrier. Given that 20% of naturally occurring boron is in the form of ^10^B isotope^42^, neutron-induced radiation damage of MTJ is a concern. Therefore, studies of the effects of gamma and neutron radiation on the properties of nanoscale MTJs are warranted.

We separated all nanoscale pMTJs studied here into three groups. The first group was exposed to gamma radiation only, the second group was exposed to a combination of gamma and thermal neutron radiation by placing the samples near the core of a nuclear reactor, and the third group of samples served as a reference that was not exposed to ionizing radiation. For the first and second groups of samples, electrical characterization of the samples was performed before and after the irradiation. For the third group of samples, two rounds of electrical characterization separated by a 6 month interval were performed in order to verify temporal stability of the samples. Electrical characterization of the samples included measurements of TMR as well as measurements of pMTJ switching by magnetic field applied perpendicular to the sample plane and by current applied to the pMTJ.

The first group of the devices consisting of 23 circular and 28 elliptical pMTJs was exposed to 2.14 kGy/h(H_2_O) gamma radiation for a total dosage of 147 kGy(SiO_2_)^43^, as described in Methods. Post-irradiation electrical characterization of this group of samples was done immediately following the exposure of the samples to gamma radiation. The second group of devices consisting of 54 circular and 66 elliptical pMTJs was exposed to radiation generated by the UC Irvine TRIGA reactor for a period of 8 hours (see Methods). The reactor generates mixed radiation consisting of thermal neutrons, gamma radiation, and high energy beta radiation. The low linear energy transfer (LET) gamma radiation dose was approximately 40 kGy/h(H_2_O) while the thermal neutron dose was 0.8 × 10^12^ cm^−2^s^−1^. Here the gamma TID of 300 kGy is 2 orders of magnitude greater than the 3 kGy immunity requirements for the Europa Clipper Mission^30^. Following the reactor irradiation, the pMTJ samples were removed from the reactor core and placed in a shielded lead cave to allow the radioactive isotopes generated in the samples to decay. After a sufficiently long time (6 months) for safe handling, post-irradiation electrical characterization of the samples was performed. At the time of post irradiation characterization, the radiation dose at the surface of the samples was 0.9 mrem/h. The residual radioactivity was primarily due to decay of ^182^Ta generated by neutron irradiation from the naturally occurring ^181^Ta present in the pMTJ leads. Finally, a group of reference samples consisting of 16 elliptical pMTJs was not exposed to any irradiation. The electrical properties of these samples were measured in the beginning and end of the same 6 month time span as the TRIGA irradiated samples.

### Field switching experiments

For all devices, the TMR and magnetic field switching characteristics were determined by means of resistance versus out-of-plane magnetic field measurements. The pMTJ devices are fabricated with macroscopic contact pads attached to the top and bottom of each individual nanoscale pMTJ. For electrical measurements, the pads are contacted by an electrical probe and resistance of the pMTJ is measured as a function of magnetic field applied perpendicular to the sample plane using a small probe current of 5 μA. Example data for an elliptical nanoscale pMTJ before and after TRIGA irradiation are shown in Fig. 1(b). Magnetic field switches the device between the low resistance state *R*_P_ corresponding to parallel (P) alignment of magnetic moments of the free and SAF layers and the high resistance state *R*_AP_ corresponding to antiparallel (AP) alignment of the magnetic moments. Since the SAF layer is designed to be stable against moderate magnetic fields, the observed hysteretic resistance switching results from magnetization reversal of the free layer. The TMR value is given by

1 $${\rm{TMR}}=\frac{{R}_{{\rm{AP}}}-{R}_{{\rm{P}}}}{{R}_{{\rm{P}}}}\times \mathrm{100 \% }.$$

**Figure 2:**
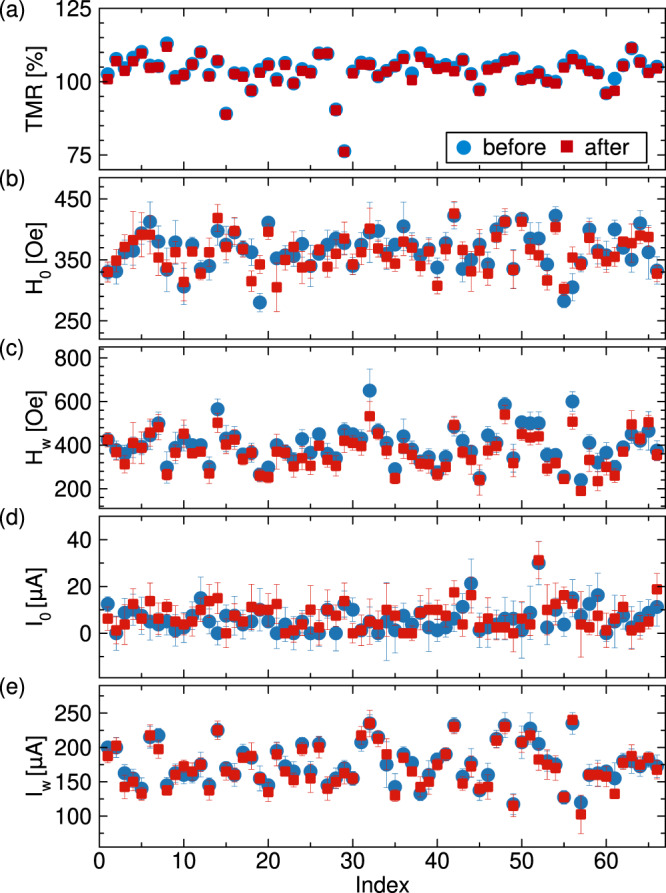
Effect of extreme ionizing radiation on pMTJ properties. Characteristics of 66 elliptical pMTJs before and after TRIGA (neutron + gamma) irradiation. **(a)** TMR, **(b)** field hysteresis loop center *H*_0_, **(c)** field hysteresis loop width *H*_w_, **(d)** current hysteresis loop center *I*_0_, and **(e)** current hysteresis loop width *I*_w_.

TMR measured before and after TRIGA irradiation is shown in Fig. 2(a) for 66 elliptical pMTJ devices.

Figure 1(b) displays multiple successive resistance versus field hysteresis loops revealing that the fields at which resistance switching takes place are different for each loop. The reason for this loop-to-loop variation is thermally activated stochastic character of the free layer switching^44,45^. The two stable states of magnetization of the free layer (up and down in Fig. 1(a)) are separated by an energy barrier arising from perpendicular magnetic anisotropy of the free layer. Thermal fluctuations lead to stochastic assisted switching of magnetization over the barrier^44–47^. As a result, switching takes place at a different magnetic field value in each hysteresis loop forming a statistical distribution of fields for P → AP and AP → P switching. We define the high (low) switching field ${H}_{{\rm{s}}}^{{\rm{high}}}$ (${H}_{{\rm{s}}}^{{\rm{low}}}$) as the median field of the P → AP (AP → P) switching distribution. The hysteresis loop width is then given by ${H}_{{\rm{w}}}={{\rm{H}}}_{{\rm{s}}}^{{\rm{h}}{\rm{i}}{\rm{g}}{\rm{h}}}-{{\rm{H}}}_{{\rm{s}}}^{{\rm{l}}{\rm{o}}{\rm{w}}}$ and the loop center is ${H}_{0}=({H}_{{\rm{s}}}^{{\rm{high}}}+{H}_{{\rm{s}}}^{{\rm{low}}})\mathrm{/2}$. The non-zero value of *H*_0_ in Fig. 1(b) arises from residual dipolar stray field produced by the SAF layer.

The effect of TRIGA irradiation on *H*_w_ and *H*_0_ for the set of 66 elliptical devices is shown in Fig. 2(b,c), respectively. The “error bars” in these figures represent the width of the thermal spread in the values of *H*_w_ and *H*_0_ as discussed in Methods. *H*_w_ is a measure of the thermal stability of the pMTJ. A significant irradiation-induced reduction of *H*_w_ would render the STT-MRAM element nonoperational.

### Spin transfer torque switching experiments

Directly after field switching measurements for each pMTJ, the spin transfer torque switching for that pMTJ was measured. The pMTJ was first prepared into the AP state by applying external field. The field was then reduced to *H*_0_, as determined from field switching. This process places the pMTJ in the center of the bistable region, the region in field space where both P and AP states coexist, see region spanned by *H*_w_ in Fig. 1(b). The current-induced switching characteristics of the pMTJs were then determined by sweeping the applied direct current at fixed *H*_0_ and measuring the change in device resistance due to TMR. Figure 1(c) shows an example of resistance versus current hysteresis loop demonstrating current-induced spin transfer torque switching of magnetization of the free layer between the P and AP states. From these data, we obtain positive and negative switching currents, ${I}_{{\rm{s}}}^{{\rm{pos}}}$ and ${I}_{{\rm{s}}}^{{\rm{neg}}}$ that are the median values of statistical distributions of the switching currents over multiple successive resistance versus current hysteresis loops. The current switching loop width is then given by ${I}_{{\rm{w}}}={{\rm{I}}}_{{\rm{s}}}^{{\rm{p}}{\rm{o}}{\rm{s}}}-{{\rm{I}}}_{{\rm{s}}}^{{\rm{n}}{\rm{e}}{\rm{g}}}$ and the current switching loop center is ${I}_{0}=({I}_{{\rm{s}}}^{{\rm{pos}}}+{I}_{{\rm{s}}}^{{\rm{neg}}})\mathrm{/2}$. The effect of TRIGA irradiation on *I*_w_ and *I*_0_ for the set of 66 elliptical devices is shown in Fig. 2(d,e), respectively. The “error bars” in these figures represent the width of the thermal spread in the values of *I*_w_ and *I*_0_ as discussed in Supplementary Material note S1.

### *In-situ* switching experiments

**Figure 3:**
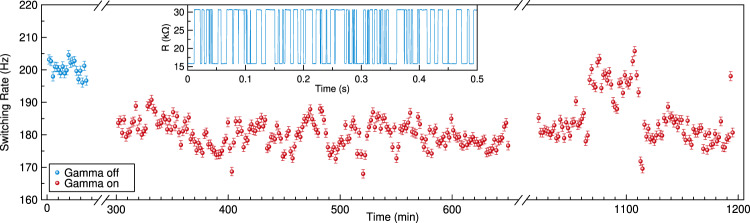
Thermally activated switching in gamma ray environment. Superparamagnetic switching rate of free layer. Blue points indicate switching rate in absence of radiation. Red points indicate switching rate in presence of radiation. Note twice broken x-axis. Inset: example time domain data of random telegraph noise.

In order to study the effects of gamma irradiation on the dynamics of thermally-activated switching of the pMTJ free layer^48^, we utilize a circular pMTJ with a thicker MgO layer and a superparamagnetic free layer^47^, where the free layer stochastically switches between the P and AP states at a characteristic rate of a few hundred Hz. The superparamagnetic free layer is a result of reduced perpendicular magnetic anisotropy such that the energy barrier for switching is comparable to the thermal energy^49^. This reduction of perpendicular anisotropy is achieved via increasing the free layer thickness above 1.6 nm and thereby bringing the free layer magnetization near transition from the out-of-plane to the in-plane equilibrium orientation. Due to TMR, as the free layer switches between the P and AP states, the device resistance displays random telegraph noise (RTN)^50,51^, which allows us to collect data on the thermally activated switching rates by measuring time dependence of the sample resistance. As we cannot manipulate external field in the gamma chamber, we use spin transfer torque from a direct current bias to tune to the center of the bistable region defined by equal dwell times in P and AP states^52^ as discussed in the Supplementary Material note S2. Example time domain RTN data are shown in the inset of Fig. 3. The switching rate measured as a function of time is shown in Fig. 3. The switching rate is monitored in the gamma chamber with both the irradiation off and on. The results show that the thermally activated switching rate varies by about 10% during the gamma radiation experiment.

The switching rate *w* of superparamagnetic MTJs is governed by the Arrhenius law^53^ $w={w}_{0}\exp (-\Delta E/{k}_{{\rm{B}}}T)$, where Δ*E* is the anisotropy energy barrier and ${w}_{0}(=\mathrm{1/}\tau )$ is the attempt frequency (inverse attempt time). One would expect any possible radiation induced switching to add to the thermally activated switching, resulting in an increase in the switching rate. We observe a small effect in the opposite direction. As the switching rate is exponentially sensitive to temperature, the 10% change can most likely be attribute to a small change in pMTJ temperature as described in Methods.

## Discussion

The results of the samples studied using the TRIGA nuclear reactor, shown in Fig. 2, reveal that extreme TID of gamma and neutron irradiation has negligible impact on all key properties of the set of 66 elliptical nanoscale pMTJs: TMR, *H*_0_, *H*_w_, *I*_0_, and *I*_w_. Quantitative analysis given in Supplementary Material note S1 shows that ensemble averages of irradiation-induced changes in these parameters do not exceed one standard deviation of these changes over the ensemble, with the exception of *H*_w_ for the elliptical pMTJs whose observed change is slightly larger than one standard deviation; however, we find that apparent irradiation-induced changes in *H*_w_ and *I*_w_ in all cases do not exceed the thermal spread of these parameters, ie. we do not observe any changes in parameter greater than our measurement error. These results allow us to conclude that changes of all key parameters of nanoscale pMTJs induced by the extreme radiation environment of the TRIGA nuclear reactor are not statistically significant and will have negligible impact on STT-MRAM performance.

Furthermore, we find that TID irradiation had negligible impact on all crucial STT-MRAM operation parameters for all the other sample sets: TRIGA reactor irradiated circular pMTJs, gamma ray irradiated circular and elliptical pMTJs, as well as the control sample elliptical pMTJs that were not exposed to any irradiation. These results are summarized in Supplementary Material note S1. In all of these samples, the ensemble average of irradiation-induced changes in TMR, *H*_0_, *H*_w_, *I*_0_, and *I*_w_ are less than one standard deviation. Additionally, the thermally dependent parameters governing STT-MRAM stability and switching characteristics, *H*_w_ and *I*_w_, are unchanged within the measurement error. These results allow us to conclude that pMTJs are immune to the effects of extreme total ionizing doses of irradiation.

In summary, our work shows that nanoscale perpendicular MTJs suitable for use in STT-MRAM applications are robust to the effects of harsh ionizing radiation. We subjected devices to extreme total dose of either gamma irradiation or gamma plus thermal neutron irradiation. The tunneling magnetoresistance, field switching, and current induced spin transfer torque switching characteristics of the pMTJs showed negligible changes after the irradiation. Furthermore, the thermally activated pMTJ switching rate was nearly unchanged under *in situ* gamma irradiation, indicating that transient effects due to gamma radiation do not affect the pMTJ switching process. This suggests that nanoscale pMTJs may find use in radiation-hard neuromorphic computing^54^.

## Methods

### Irradiation considerations

Gamma radiation was provided using an in-house 5,000 Ci Cs-137 gamma cell at a dose rate of 2.14 kGy/h water equivalent dose, which is approximately equivalent to 1.96 kGy/h in silica using a conversion factor of 0.916^43^. The accumulated gamma dose to the chips was 160 kGy (160,000 J energy deposited per kg mass) to water or 147 kGy(SiO_2_). After irradiation of the samples to reach the total dose, the samples were removed from the gamma cell and taken for post irradiation characterization.

A mixed radiation field of low linear energy transfer (LET) radiation from gamma and high energy beta as well as neutrons was provided using the UC Irvine TRIGA reactor. The samples were lowered into an irradiation position in the Lazy Susan compartment of the reactor core where the LET dose was approximately 40 kGy/h dose to water and the thermal neutron dose was 0.8 × 10^12^ cm^−2^ s^−1^. The samples were irradiated for 8 hours and were subsequently removed from the core and placed in a shielded lead cave to allow the radioactive isotopes to decay. After a sufficient time (6 months) for safe handling, the samples were taken for post irradiation characterization. At the time of post irradiation characterization, the radiation dose at the surface of the samples was 0.9 mrem/h. The remaining radioactivity was primarily due to ^182^Ta.

### Thermally activated switching in gamma ray environment

We study thermally driven stochastic switching in the gamma ray environment by means of real time resistance measurements of a superparamagnetic perpendicular magnetic tunnel junction (pMTJ). The superparamagnetic state is achieved when the perpendicular magnetic anisotropy energy barrier is not too large compared to the thermal energy. Such a system displays random telegraph noise (RTN) as the free layer magnetization stochastically switches due to thermal activation between the parallel (lower resistance) and anti-parallel (higher resistance) states with respect to the reference layer. The switching rate *w* of superparamagnetic MTJs is governed by Arrhenius law^53^

2 $$w={w}_{0}\exp (-\Delta E/{k}_{{\rm{B}}}T),$$

where Δ*E* is the anisotropy energy barrier and *w*_0_ is the attempt frequency. We exploit the exponential sensitivity of the switching rate to changes of the pMTJ properties to determine the impact of gamma radiation on the state of the pMTJ.

The pMTJ chip is attached to an aluminum sample holder via thermal paste. A resistance thermometer device (RTD) and heater are also securely mounted to the sample holder. We use PID control to keep the sample holder at constant elevated temperature *T* = 300 K throughout the experiment. The temperature is stabilized above the average room temperature of *T*_room_ ≈ 295 K in order to ensure stable operation of the PID temperature control with ambient environment serving as a heat sink. However, we cannot measure and control the temperature of the pMTJ chip surface directly; therefore the temperature of the pMTJ can vary slightly from the sample holder due to non-uniform heating of objects inside the chamber by the gamma radiation. As shown in Fig. 3, we observe a reduction of the RTN switching rate of ≈10% with the gamma chamber activated; however, this effect is very small considering the exponential sensitivity of the switching rate to temperature. The small change in switching rate is most likely due to the small difference in temperature of the pMTJ chip surface when the gamma rays are on and off. In the situation where the sample holder heats more during irradiation, the pMTJ temperature can be lower than *T* = 300 K and lead to the observed reduction of switching rate. The relatively poor thermal conductivity of the thermally oxidized Si substrate can support a temperature gradient between the pMTJ on its surface and sample holder. In Supplementary Material note S3, using the Arrhenius law we estimate a reduction of pMTJ temperature of Δ*T* < 2 K from the sample holder temperature of 300 K would account for the observed effect. Measuring this temperature difference would require lithographic patterning of a thermometer on the chip surface right next to the pMTJ, which is beyond the scope of our current study. The observed absence of large variations of the RTN switching rate during gamma radiation indicates the insensitivity of the pMTJ switching to gamma irradiation.

## Data Availability

All data supporting the findings of this study are available within the article and the Supplementary Information file, or are available from the corresponding author on reasonable request.

## Supplemenatry information

Supplemenatry information.
