# Supplementary material for: Immunity of nanoscale magnetic tunnel junctions with perpendicular magnetic anisotropy to ionizing radiation
Source: Sci Rep. 2020 Jun 23;10:10220. doi: 10.1038/s41598-020-67257-2 (PMC7311406; doi:10.1038/s41598-020-67257-2)
Supplement: Supplementary file 1 — Supplemenatry information. [file 41598_2020_67257_MOESM1_ESM.pdf]

Supplementary Material for Immunity of nanoscale magnetic tunnel junctions with perpendicular magnetic anisotropy to ionizing radiation

Eric Arturo Montoya, Jen-Ru Chen, Randy Ngelale, Han Kyu Lee, Hsin-Wei Tseng, Lei Wan, En Yang, Patrick Braganca, Ozdal Boyraz, Nader Bagherzadeh, Mikael Nilsson, and Ilya N. Krivorotov

S1 Irradiation results

Due to the stochastic, thermally-activated character of both the field- and current-induced switching, the switching fields and switching currents exhibit a statistical distribution of values in the same pMTJ device. In our analysis below, the high and low switching fields (left and right coercive fields), $H_{\mathrm{s}}^{\mathrm{high}}$ and $H_{\mathrm{s}}^{\mathrm{low}}$, and positive and negative switching currents, $I_{\mathrm{s}}^{\mathrm{pos}}$ and $I_{\mathrm{s}}^{\mathrm{neg}}$, are the median values of these statistical distributions. The field loop width is given by $H_{\mathrm{w}} = H_{\mathrm{s}}^{\mathrm{high}} - H_{\mathrm{s}}^{\mathrm{low}}$ and the loop center is given by $H_0 = \left(H_{\mathrm{s}}^{\mathrm{high}} + H_{\mathrm{s}}^{\mathrm{low}}\right)/2$. Similarly, the current switching loop width is given by $I_{\mathrm{w}} = I_{\mathrm{s}}^{\mathrm{pos}} - I_{\mathrm{s}}^{\mathrm{neg}}$ and the current switching loop center is given by $I_0 = \left(I_{\mathrm{s}}^{\mathrm{pos}} + I_{\mathrm{s}}^{\mathrm{neg}}\right)/2$. To visualize and characterize the thermally induced spread in switching fields and switching currents we use "error bars" that are represented by the interquartile range (IQR) of the measured statistical distributions given by

$$IQR = Q_3 - Q_1, \tag{S1}$$

where is Q_1 is the first quartile and Q_3 is the third quartile. In the case of n switching events, Q_1 is given by the median of the smallest $n/2$ switching events and Q_3 is given by the median of the largest $n/2$ switching events. The characteristic spread in the field switching loop width for a given device is $\delta H_{\mathrm{w}} = \left(\left(\delta H_{\mathrm{s}}^{\mathrm{high}}\right)^2 + \left(\delta H_{\mathrm{s}}^{\mathrm{low}}\right)^2\right)^{1/2}$, where the characteristic spreads in switching field $\delta H_{\mathrm{s}}^{\mathrm{high}}$ and $\delta H_{\mathrm{s}}^{\mathrm{low}}$ are given by the IQR as described above. The characteristic spread in field loop center is $\delta H_0 = \delta H_{\mathrm{w}}/2$. Similarly, the characteristic spread in the current switching loop width is $\delta I_{\mathrm{w}} = \left(\left(\delta I_{\mathrm{s}}^{\mathrm{pos}}\right)^2 + \left(\delta I_{\mathrm{s}}^{\mathrm{neg}}\right)^2\right)^{1/2}$. The characteristic spread in current switching loop center is $\delta I_0 = \delta I_{\mathrm{w}}/2$. The total "error bars" plotted in Fig. 2 and Fig. S1-S4 are given by $\pm$ the characteristic spread for the corresponding value.

We monitor the effects of irradiation on a set of pMTJ parameters X important for the operation of STT-MRAM, where ($X = \mathrm{TMR}, H_0, H_{\mathrm{w}}, I_0, I_{\mathrm{w}}$). The irradiation-induced change ΔX in the mean value of the parameter X for a set of pMTJ devices is shown in Table 1. This table also shows standard deviation σ_X of irradiation-induced changes in X calculated for this set of pMTJ devices.

Specifically, the irradiation-induced change in the mean value of X is defined as

$$\Delta X = \frac{1}{N}\sum_{i=1}^{N}\left(X^{after,i} - X^{before,i}\right), \tag{S2}$$

where i is the pMTJ device index and N is total the number of pMTJ devices in the set. For example, a negative value for the change in the width of the field switching loop, given by $\Delta H_{\mathrm{w}} = (1/N)\sum_{i=1}^{N}(H_{\mathrm{w}}^{after,i} - H_{\mathrm{w}}^{before,i})$, would correspond to a general trend for the narrowing of the loop, and thus a reduction in the coercivity, after a particular irradiation.

The standard deviation σ_X, defined as

$$\sigma_X = \left(\frac{1}{N-1}\sum_{i=1}^{N}\left(\left(X^{after,i} - X^{before,i}\right) - \Delta X\right)^2\right)^{\frac{1}{2}}, \tag{S3}$$

gives a measure of the error in detecting irradiation-induced change in parameter X.

As the field and current induced switching processes are thermally assisted, we would additionally like to compare irradiation-induced changes ΔH_{w} and ΔI_{w} to the thermal spread of these parameters δH_{w} and δI_{w}. The thermal spread of the parameters δX are an indication of the experimental error in determining the value of parameter X as the error is dominated by thermal fluctuations.

Table 1. Summary of irradiation effects on nanoscale pMTJs

Shape	Irradiation	N	ΔTMR %	σ_{TMR} %	ΔH_0 Oe	σ_{H_0} Oe	ΔH_{w} Oe	$\sigma_{H_{\mathrm{w}}}$ Oe	$\varepsilon_{H_{\mathrm{w}}}$ Oe	ΔI_0 μA	σ_{I_0} μA	ΔI_{w} μA	$\sigma_{I_{\mathrm{w}}}$ μA	$\varepsilon_{I_{\mathrm{w}}}$ μA
Circle	Gamma	23	-0.1	0.6	-9	20	-4	43	60	0	3	0	3	5
Ellipse	Gamma	28	-0.1	0.7	0	19	-2	17	44	3	5	-3	7	11
Circle	TRIGA	54	-0.1	0.4	-11	25	-25	41	60	0	2	-1	4	6
Ellipse	TRIGA	66	-0.6	0.6	-5	21	-32	30	40	1	6	-4	8	13
Ellipse	None	16	0.1	2.5	9	18	33	36	40	1	5	7	10	12

Specifically, we compare ΔH_{w} to the average of thermal spread in the field loop width over the set of pMTJ devices:

$$\varepsilon_{H_{\mathrm{w}}} = \frac{1}{2N}\sum_{j=1}^{2N} \delta H_{\mathrm{w}}^{j}, \tag{S4}$$

where the index j includes individual measurements before and after the irradiation and thus the total measurements is twice the pMTJ device set size. Similarly, we calculate the average thermal spread in the current switching loop width I_{w} as

$$\varepsilon_{I_{\mathrm{w}}} = \frac{1}{2N}\sum_{j=1}^{2N} \delta I_{\mathrm{w}}^{j}, \tag{S5}$$

The values for $\varepsilon_{H_{\mathrm{w}}}$ and $\varepsilon_{I_{\mathrm{w}}}$ are also tabulated in Table 1.

The before and after irradiation results for TMR, H_0, H_{w}, I_0, and I_{w} are shown in Fig. S1 for the gamma irradiated circular pMTJs, Fig. S2 for the gamma irradiated elliptical pMTJs, and Fig. S3 for the TRIGAirradiated circular pMTJs. The before and after 6 months waiting time for the non-irradiated control elliptical pMTJs are shown Fig. S4. For the vast majority of the devices the before/after medians of TMR, H_0, H_{w}, I_0, and I_{w} overlap within one IQR, which is a visual indication that the extreme doses of ionizing radiation had negligible permanent effect on the pMTJs. This can also be seen numerically in Table 1 that shows that ensemble averages of irradiation-induced changes in all these parameters do not exceed one standard deviation of these changes over the ensemble in all experiments with the exception of ΔH_{w} for the elliptical pMTJs. In this case, the observed change in $\Delta H_{\mathrm{w}} = -32$ Oe is slightly larger than one standard deviation $\sigma_{H_{\mathrm{w}}} = 30$ Oe; however, we find the apparent change in H_{w} is still less than the experimental error in determining H_{w} due to thermal fluctuations $\varepsilon_{H_{\mathrm{w}}} = 40$ Oe.

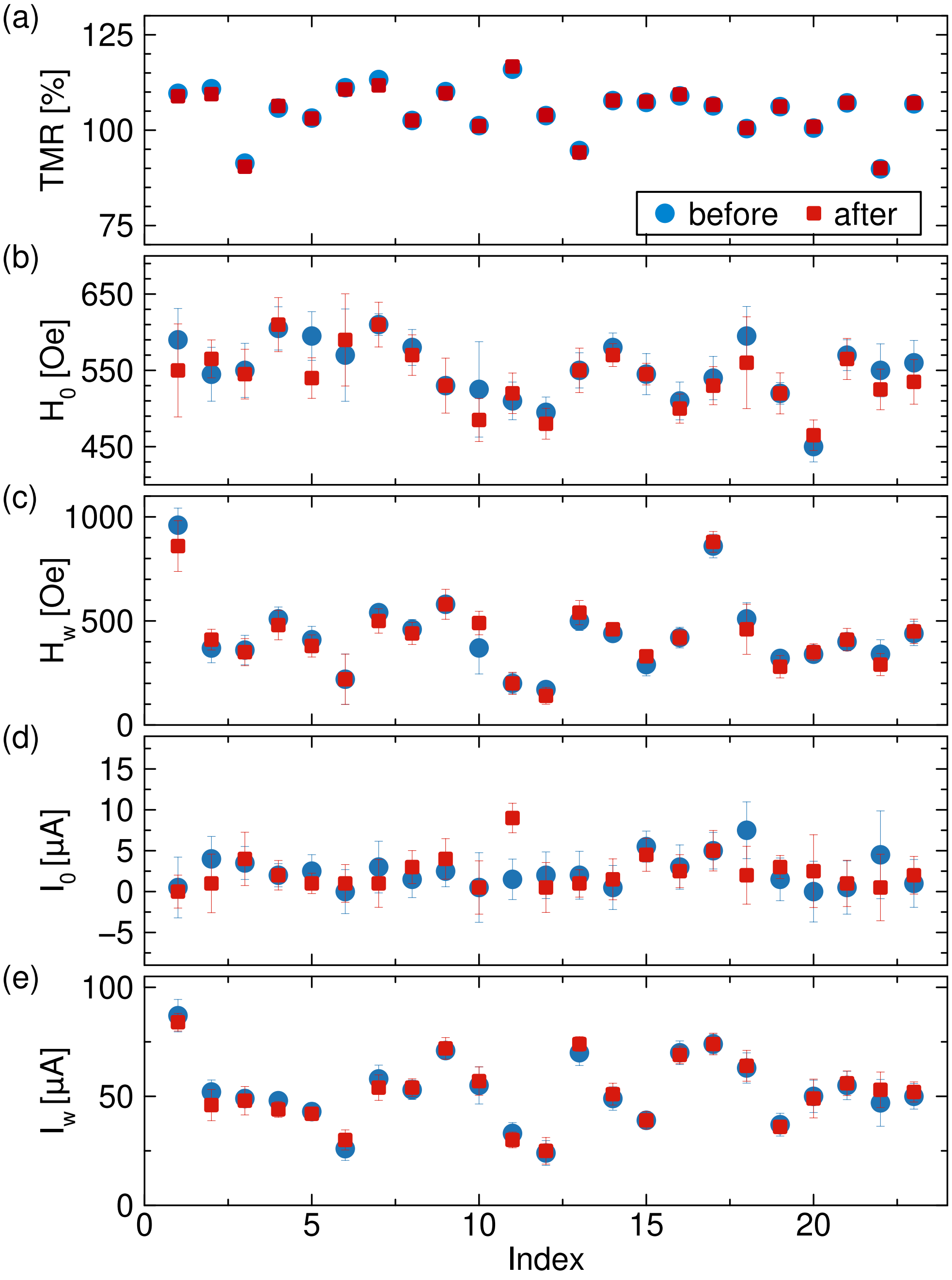

Figure S1. Before and after gamma irradiation results for 23 circular pMTJ devices. (a) TMR, **(b)** field hysteresis loop center H_0, **(c)** field hysteresis loop width H_w, **(d)** current hysteresis loop center I_0, and **(e)** current hysteresis loop width I_w.

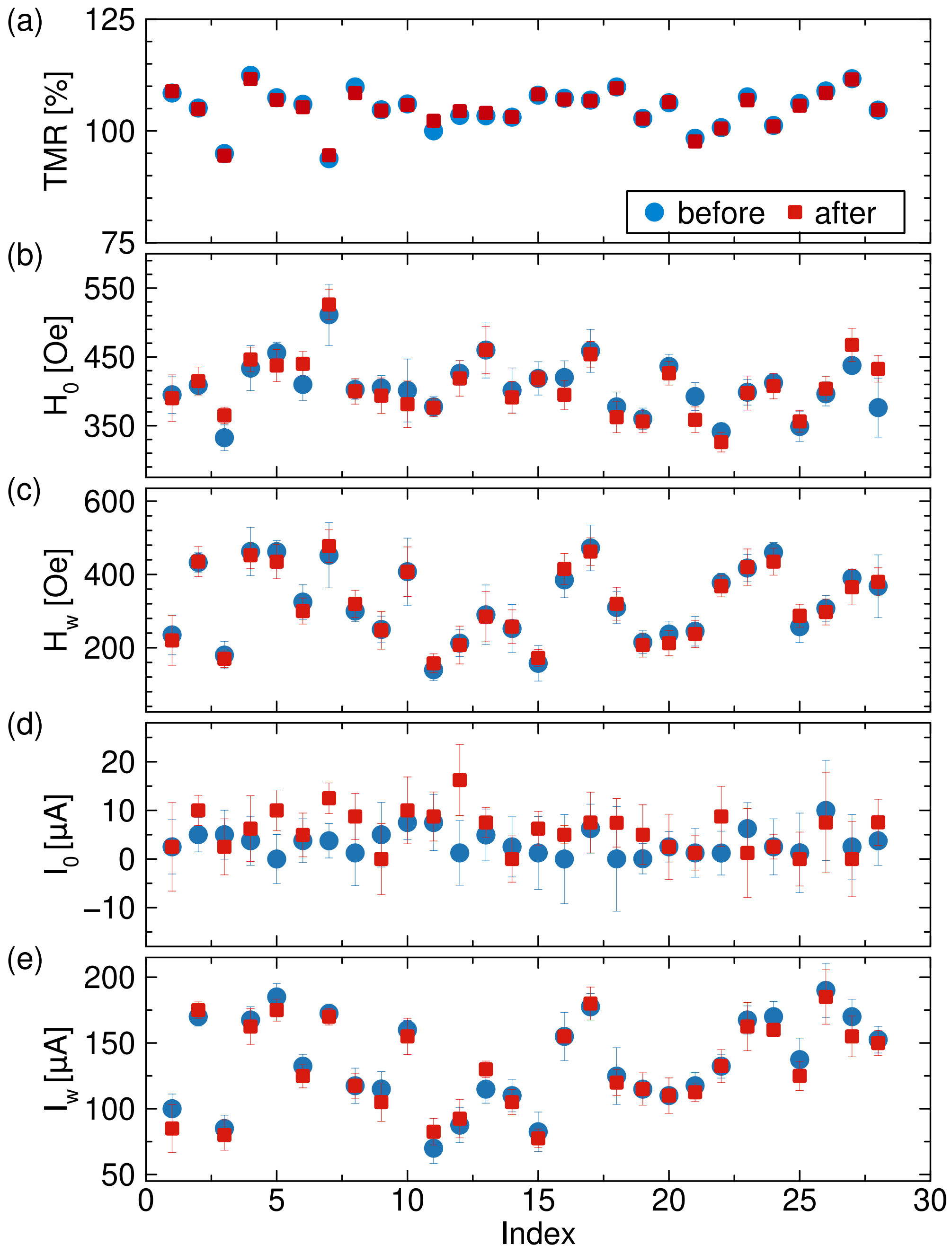

Figure S2. Before and after gamma irradiation results for 28 elliptical devices. **(a)** TMR, **(b)** field hysteresis loop center H_0, **(c)** field hysteresis loop width H_w, **(d)** current hysteresis loop center I_0, and **(e)** current hysteresis loop width I_w.

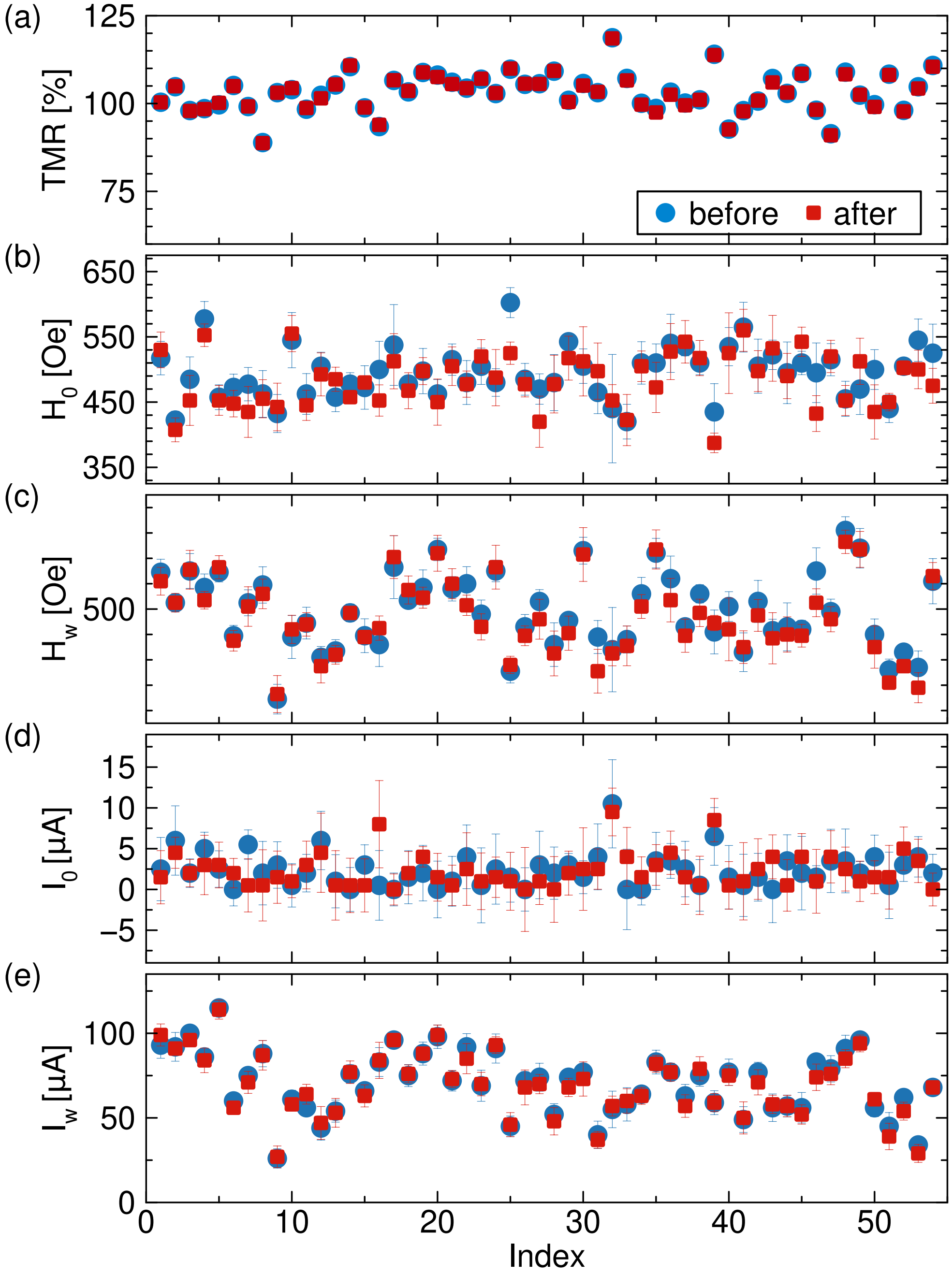

Figure S3. **Before and after TRIGAirradiation results for 54 circular devices. (a)** TMR, **(b)** field hysteresis loop center H_0, **(c)** field hysteresis loop width H_w, **(d)** current hysteresis loop center I_0, and **(e)** current hysteresis loop width I_w.

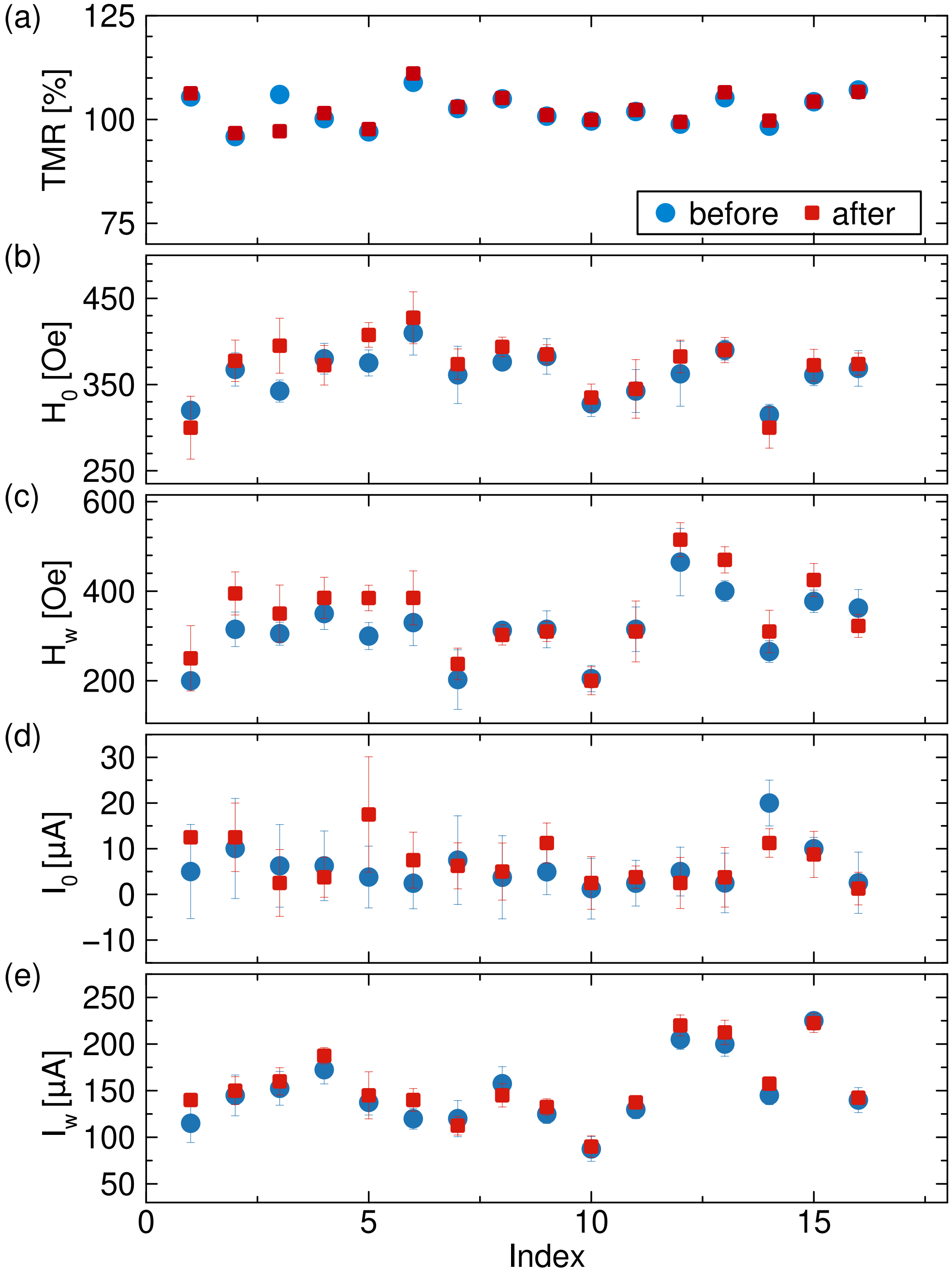

Figure S4. Before and after 6 months in non-radiation environment results for 16 elliptical devices. **(a)** TMR, **(b)** field hysteresis loop center H_0, **(c)** field hysteresis loop width H_w, **(d)** current hysteresis loop center I_0, and **(e)** current hysteresis loop width I_w.

S2 Tuning superparamagnetic pMTJ to center of bistable region using spin transfer torque

We use random telegraph noise (RTN) measurements to characterize thermally activated switching of pMTJs in the gamma ray environment. Traditionally RTN measurements are performed in an applied external field that is used to compensate for reference layer stray fields and tune the MTJ to the center of the bistable region[1,2]. In the center of the bistable region, the dwell time in the P and AP states are equal, $\tau_\mathrm{P} = \tau_\mathrm{AP}$. We define the switching rate w_x for a given state $(x = \mathrm{P}, \mathrm{AP})$, as the inverse of the dwell time of that state such that $w_\mathrm{P}\,(\equiv 1/\tau_\mathrm{P})$ is the switching rate from P $\rightarrow$ AP and $w_\mathrm{AP}\,(\equiv 1/\tau_\mathrm{AP})$ from AP $\rightarrow$ P. In the

the center of the bistable region $w_P = w_{AP}$; therefore, the switching rate for the pMTJ can be defined as $w = w_P = w_{AP}$.

Since our gamma irradiation chamber lacks *in situ* magnetic field tuning capabilities, we use an alternative method of biasing the sample to the center of the bistable region. Specifically, we apply spin transfer torque to tune the pMTJ into the center of the bistable region[3]. Fig. S5 shows the switching rates w_P and w_{AP} as a function of applied bias current I. The switching rates display an exponential dependence on the bias current (logarithmic y-axis)[3]. A slower switching rate, longer dwell time, indicates that state is more stable. For example in Fig. S5, reducing the magnitude of I biases the pMTJ into the P state as indicated by reduced w_P and increased w_{AP}. Increasing the magnitude of I biases the pMTJ toward the AP state as indicated by increased w_P and decreased w_{AP}. We use bias current of $I = -4.98\,\mu$A for this sample to tune the pMTJ into the center of the bistable region such that $w_P = w_{AP}$.

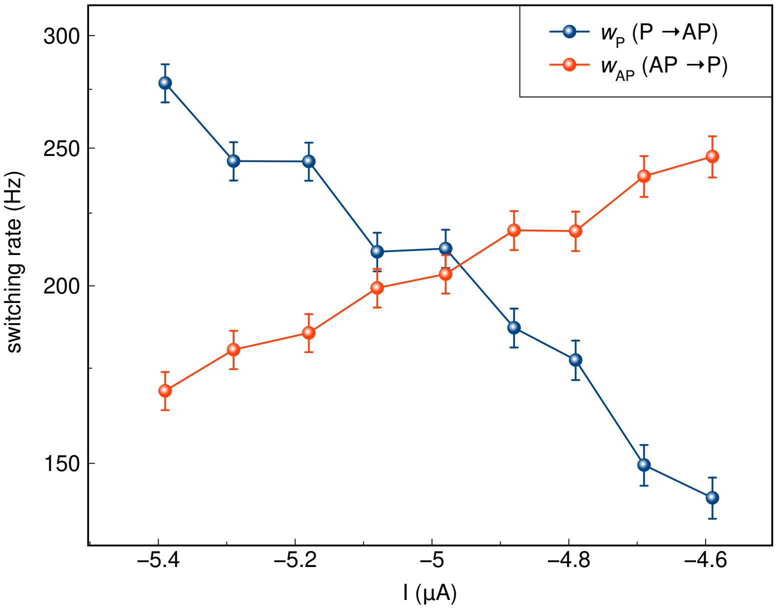

Figure S5. Switching rates w_P and w_{AP} as a function of bias current I applied to superparamagnetic pMTJ. The condition $w_P = w_{AP}$ indicates the center of the bi-stable region where the pMTJ has equal probability of being in the P and AP states.

S3 Estimate of temperature change of pMTJ in gamma chamber for thermally activated switching experiment

The Arrhenius law[4] can be used to estimate the change in temperature required for the observed deviation of the switching rate w of the superparamagnetic MTJ in the random telegraph noise experiment. The Arrhenius law written in terms of switching rate is,

$$w = w_0 \exp\left(-\Delta E / k_B T\right), \tag{S6}$$

where ΔE is the magnetic anisotropy energy barrier and $w_0 \approx 1\,\mathrm{GHz}$ is the attempt frequency[5,6]. For the observed switching rate in the absence of gamma irradiation $w_{(300K)} = 200\,\mathrm{Hz}$, we can estimate the effective energy barrier expressed as a temperature of $\Delta E / k_B = 4630\,\mathrm{K}$. The average switching rate with the gamma irradiation on, $w_\gamma = 182.2\,\mathrm{Hz}$, could be caused by a temperature change of the pMTJ of $\Delta T < 2\,\mathrm{K}$.

References

1. Jiang, L. *et al.* Low-frequency magnetic and resistance noise in magnetic tunnel junctions. *Phys. Rev. B* **69**, 054407, DOI: 10.1103/PhysRevB.69.054407 (2004).

2. Costanzi, B. N. & Dahlberg, E. D. Emergent 1 / f Noise in Ensembles of Random Telegraph Noise Oscillators. *Phys. Rev. Lett.* **119**, 097201, DOI: 10.1103/PhysRevLett.119.097201 (2017).

3. Krivorotov, I. N. *et al.* Temperature Dependence of Spin-Transfer-Induced Switching of Nanomagnets. *Phys. Rev. Lett.* **93**, 166603, DOI: 10.1103/PhysRevLett.93.166603 (2004).

4. Van Der Ziel, A. On the noise spectra of semi-conductor noise and of flicker effect. *Physica* **16**, 359–372, DOI: 10.1016/0031-8914(50)90078-4 (1950).

5. Suh, H.-J. *et al.* Attempt frequency of magnetization in nanomagnets with thin-film geometry. *Phys. Rev. B* **78**, 064430, DOI: 10.1103/PhysRevB.78.064430 (2008).

6. Lo Conte, R. *et al.* Spin-orbit torque-driven magnetization switching and thermal effects studied in Ta\CoFeB\MgO nanowires. *Appl. Phys. Lett.* **105**, 122404, DOI: 10.1063/1.4896225 (2014).
